# Identifying and Characterizing Conveyor Belt Longitudinal Rip by 3D Point Cloud Processing

**DOI:** 10.3390/s21196650

**Published:** 2021-10-07

**Authors:** Shichang Xu, Gang Cheng, Yusong Pang, Zujin Jin, Bin Kang

**Affiliations:** 1 School of Mechatronic Engineering, China University of Mining and Technology, Xuzhou 221116, China; TB19050022B2@cumt.edu.cn (S.X.); jinzjcam@cumt.edu.cn (Z.J.); TS18050105P31@cumt.edu.cn (B.K.); 2 Shandong Zhongheng Optoelectronic Technology Co., Ltd., Zaozhuang 277000, China; 3 Faculty Mechanical, Maritime and Materials Engineering, Delft University of Technology, 2628 Delft, The Netherlands; Y.Pang@tudelft.nl

**Keywords:** longitudinal rip, 3D point cloud, clustering process, principal component analysis (PCA)

## Abstract

Real-time and accurate longitudinal rip detection of a conveyor belt is crucial for the safety and efficiency of an industrial haulage system. However, the existing longitudinal detection methods possess drawbacks, often resulting in false alarms caused by tiny scratches on the belt surface. A method of identifying the longitudinal rip through three-dimensional (3D) point cloud processing is proposed to solve this issue. Specifically, the spatial point data of the belt surface are acquired by a binocular line laser stereo vision camera. Within these data, the suspected points induced by the rips and scratches were extracted. Subsequently, a clustering and discrimination mechanism was employed to distinguish the rips and scratches, and only the rip information was used as alarm criterion. Finally, the direction and maximum width of the rip can be effectively characterized in 3D space using the principal component analysis (PCA) method. This method was tested in practical experiments, and the experimental results indicate that this method can identify the longitudinal rip accurately in real time and simultaneously characterize it. Thus, applying this method can provide a more effective and appropriate solution to the identification scenes of longitudinal rip and other similar defects.

## 1. Introduction

A belt conveyor is widely used in the industrial field and is mainly used in material transportation equipment [1,2,3,4,5]. The longitudinal rip of the belt—along the running direction caused by hard impurities’ puncture, penetration, and blocking—is one of the common faults of the belt conveyor. The identification of the longitudinal rip in real-time can avoid further extension of the rip, which may cause material leakage, conveyor damage, transport system paralysis, and even safety accidents [6,7,8,9]. As a result, many methods for identifying longitudinal rips have been proposed.

The method first used to identify the longitudinal rip was the traditional mechanical method [10,11], which indirectly identifies the longitudinal rip by detecting materials or impurities leaking through the rip, but has the obvious shortcomings in identification time. Afterwards, the non-contact identification methods based on ultrasonic [12,13], radio frequency [14,15], and electromagnetic induction [16,17], are employed in the industrial field, and these methods have decreased the identification time to a sub-second. Nonetheless, there are still deficiencies in accuracy and reliability when using these methods.

In recent years, identification methods based on image processing have been gradually developed. A method was proposed to preliminarily identify the rip by the defect information extracted from the pixels of images, such as area, slightness, and rectangle degree [18]. Another method based on infrared and visible light fusion was adopted to detect the longitudinal rip of conveyor belts [19], which improved the identification accuracy. A monitoring system was designed to identify the longitudinal rip by extracting the laser stripe skeleton and distinguishing the jump distortion [20], which has a good performance in response time. However, in some harsh environments, due to the uneven illumination and the stains attached to the belt surface, image-processing methods for pixel color often fail. Furthermore, on the belt’s surface, there are many small scratches, which have no effect on the normal running of the conveyors, but based on image processing, those scratches are often mistakenly identified as longitudinal rips, resulting false alarms and unplanned downtime to seriously affect production efficiency. Thus, an improved longitudinal rip detection method with higher accuracy, reliability, and real-time still needs to be exploited. In contrast to the image processing method, which extracts characteristics from the color or brightness of pixels in the two-dimensional images, the method based on point cloud processing deals with a set of three-dimensional point coordinates [21,22]. It means that this method can achieve more accurate data acquisition and three-dimensional measurement of object surface [23,24,25].

In this work, a novel longitudinal rip detection and characterization method based on 3D point cloud processing is proposed and demonstrated. To be specific, a binocular line laser stereo vision camera was used to obtain the point cloud data on the lower surface of the belt by a line-scanning mode. Through the convenient threshold judgments, the suspected points induced by the rips or scratches within the point cloud data could be extracted. Then, these suspected points were clustered and the clusters of scratch points were eliminated by a distance recognition mechanism. Then only the clusters of the longitudinal rips were treated as the alarm criteria to achieve the identification operation. The method in this work has the following three advantages: first, it prevents the probability of false alarm by overcoming the interferences of the uneven illumination in harsh environment and the scratches on belt surface. The identification correct rate obtained from a large amount of tests is 99.2%. Second, it has exceptional advantages in real-time, and the identification time of longitudinal rip is less than 0.04 ms. Third, the direction and maximum width of the longitudinal rip in 3D space can be determined simultaneously with high precision.

## 2. System Setup and Algorithm Flow in This Work

**Figure 1:**
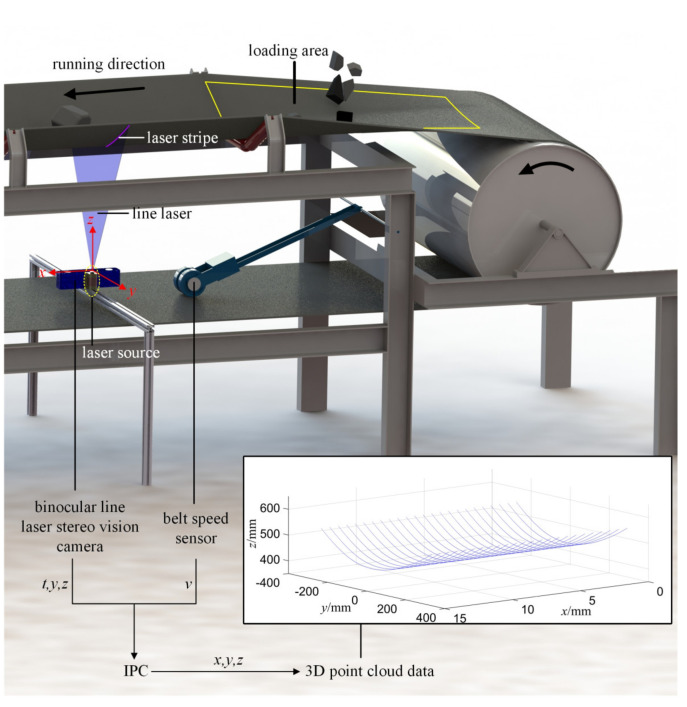
System setup and data acquisition process; IPC, industrial personal computer.

The diagram of the system setup and belt surface data acquisition process are shown in Figure 1. A data acquisition system, including a binocular line laser stereo vision camera and a belt speed sensor, was mounted near the loading area of a conveyor where the longitudinal rip was most likely to occur (90%) [14]. In this system, the camera was installed between the upper and lower belts, on which, a laser source projected a line laser with a certain fan angle on the lower surface of the upper belt (then a laser stripe could be generated on the belt surface along the belt’s width direction (the *y* direction)). The belt ran in the *x* direction and a belt speed sensor was used to measure its running speed in real time. To further explain, the operating principle of the camera was to collect spatial data by using binocular parallax theory [26]. The camera collected point data (*t_i_*, *y_j_*, *z_j_*) of about 2000 points on the laser stripe. *t_i_* was the timestamp to get these points on the *i-*th laser stripe; *y_j_* and *z_j_*, respectively, denote the coordinate values in the width and height directions of the *j-*th point on the laser stripe from left to right. Then, these point data were sent to the industrial personal computer (IPC).

The *x* coordinate value of each point can be presented as:

(1) $$x_{j}=vt_{i}\;,\;j=1,\;2,\;3,\;\cdots ,\;2000$$

where *v* presents the real-time belt speed, which can be derived from a belt speed sensor (A list of symbols in this paper with units and notes is created in Appendix A, please see Table A1). Thus, the 3D point cloud data on each laser stripe can be obtained:

(2) $$P_{j}=\left(x_{j},\;y_{j},\;z_{j}\right)\;,\;j=1,\;2,\;3,\;\cdots ,\;2000$$

As the belt runs, its lower surface will be scanned by the camera so that the 3D point cloud data will be obtained line-by-line, and the data will be applied as the raw data.

**Figure 2:**
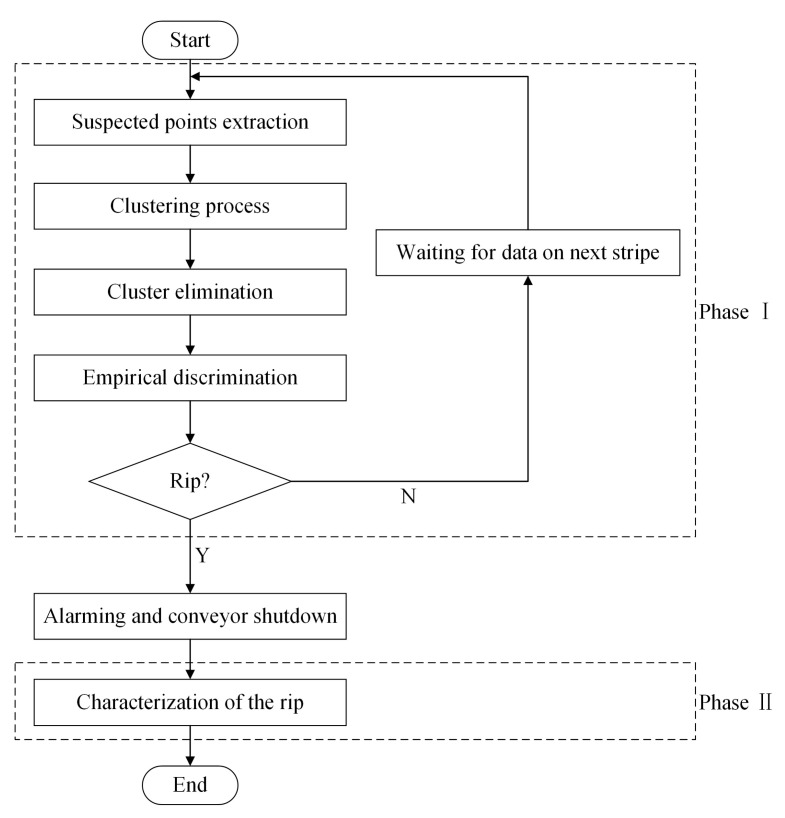
Flowchart of algorithm in this work.

The flowchart of algorithm in this work is shown in Figure 2. The function realization of the system is divided into two phases, before and after the occurrence of the longitudinal rip. The first one is the identification of the longitudinal rip. In this phase, we take the time interval of the 3D data input between the present stripe and next stripe as an identification cycle. In each cycle, the original data on the present stripe will be processed through four steps: suspected points extraction, clustering process, cluster elimination, and empirical discrimination. Specifically, at the beginning, the suspected points induced by the rips or scratches are extracted by convenient threshold judgments. Then through the clustering process, these points are classified into different clusters. Subsequently, through the elimination of clusters, points induced by the scratches, which have been completely scanned, are eliminated. Finally, the empirical discrimination based on length detection is applied to identify the longitudinal rip. Once the longitudinal rip is identified, the intelligent decisions (alarm and automatic shutdown) will be implemented. Then, the points in the rip clusters are used to further characterize the longitudinal rip in the second phase, so that the direction and maximum width of the longitudinal rip can be effectively characterized in 3D space.

## 3. Phase I: Identification of the Longitudinal Rip

As stated above, the real-time identification of the longitudinal rip includes four steps: suspected points extraction, clustering process, cluster elimination, and empirical discrimination. Each processing step will be explained in the following sections.

### 3.1. Suspected Points Extraction

**Figure 3:**
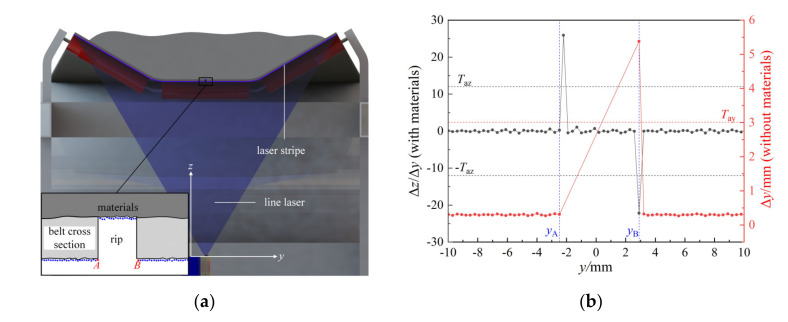
Point distribution near the rip. (**a**) The cross-section view with the line laser plane as the cutting plane, the bottom illustration is the zoom view of the middle rectangle box near the rip. (**b**) The data analysis chart for finding the suspected points (point *A* and *B* shown in (**a**)), the black dots correspond to the values of Δ*z*/Δ*y* of the points when the belt upper surface covered with materials, while the red dots correspond to the values of Δ*y* of the points when the belt upper surface covered with no materials.

Figure 3a shows the cross-section view with the line laser plane as the cutting plane, the blue dots in the zoom view are a set of 3D points collected by the camera, and the distribution of the 2000 original 3D points input in each cycle reflects the morphological information of the belt’s lower surface. Under normal circumstances, the points should be evenly distributed in *y* and *z* directions, but when there is a rip on the belt surface, the point distribution near the rip’s edges will induce abnormal jump fluctuations. Thus, by extracting the suspected points (point *A* and *B* in Figure 3a), which cause fluctuations, we can get the information of the rip edges.

The threshold judgments are used to extract the suspected points and the two cases of belt with and without materials cover are discussed separately as follow.

(1) Belt covered with materials.

When there is a rip on the belt surface, the line laser will pass through the rip and project on materials. In the vicinity of the longitudinal rip, there will be a sudden change in the *z* direction between two adjacent points. We define the change rate of point *P_j_*(*x_j_*,*y_j_*,*z_j_*) as:

(3) $$\Delta z_{j}/\Delta y_{j}=\left(z_{j}-z_{j}{}_{-1}\right)/\left(y_{j}-y_{j}{}_{-1}\right)\;,\;j=2,\;3,\;4,\;\cdots ,\;2000$$

As shown in Figure 3b, the black dots represent the change rate in *z* direction of each 3D point. The change rate of point *B* is much smaller than the conventional value and the change rate of the adjacent point on the right side of point *A* is much larger than this value. Hence, point *P_j_*(*x_j_*, *y_j_*, *z_j_*) is considered a suspected point, if it meets one of the following conditions:

(4) $$\Delta z_{j}/\Delta y_{j}<-T_{\mathrm{az}\;}\;\mathrm{or}\;\Delta z_{j+1}/\Delta y_{j+1}>T_{\mathrm{az}}$$

where *T*_az_ is the threshold of the change rate in *z* direction and determined by the following formula:

(5) $$T_{\mathrm{az}}=s_{a}b/\left(\frac{y_{\max}-y_{\min}}{2000}\right),\;s_{a}=0.3\sim 0.7$$

where *b* presents the thickness of the belt, *y*_max_ and *y*_min_ represent the largest and the smallest *y* coordinate values among the 2000 points, separately. *s*_a_ between 0.3 and 0.7 is an empirical coefficient that is obtained through a large number of experiments.

(2) Belt covered without materials.

Under the circumstance of belt covered without materials, the line laser will pass through the rip and no point will be collected in the rip area. It means that the laser stripe is interrupted by the longitudinal rip and a few 3D points are lost. In this case, we define the space in *y* direction between two adjacent points *P_j_*_-1_(*x_j_*_-1_,*y_j_*_-1_,*z_j_*_-1_) and *P_j_*(*x_j_*,*y_j_*,*z_j_*) as:

(6) $$\Delta y_{j}=y_{j}-y_{j}{}_{-1}\;,\;j=2,\;3,\;4,\;\cdots ,\;n,\;n<2000$$

As shown in Figure 3b, the red dots represent the space in *y* direction between two 3D adjacent points *P_j_*_-1_(*x_j_*_-1_, *y_j_*_-1_, *z_j_*_-1_) and *P_j_*(*x_j_*, *y_j_*, *z_j_*). We can also see that the space in the *y* direction between point *A* and *B* is much wider than the conventional value. Thus, point *P_j_*_-1_ and *P_j_* are considered to be a pair of suspected points, if the following condition is met:

(7) $$\Delta y_{j}>T_{\mathrm{ay}}$$

where *T*_ay_ is the threshold of space in *y* direction and determined by the following empirical formula:

(8) $$T_{\mathrm{ay}}=6\left(y_{\max}-y_{\min}\right)/2000$$

Combining the extraction methods proposed in case (1) and (2), the suspected points can be found in all possible application scenarios.

**Figure 4:**
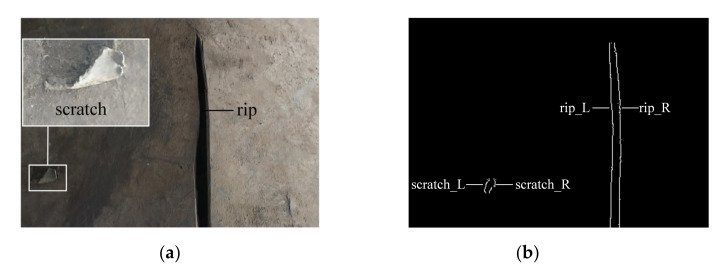
The result of suspected point extraction process. (**a**) The physical picture, the upper illustration is the zoom view of the lower rectangle box. (**b**) The in-situ result image of (**a**).

Figure 4 shows the result of the suspected point extraction process. Figure 4a shows a piece of belt where there is an obvious longitudinal rip and a tiny scratch on its surface. Figure 4b is the in-situ result image of Figure 4a. White dots are the suspected points extracted in each processing cycle. It can be seen that, not only the suspected points induced by the longitudinal rip, but also the scratches all have been extracted. Note that scratches in Figure 4a are common for the industrial conveyor belts, especially on the old belt surfaces. Therefore, in order to differentiate the rips from the scratches, we need to eliminate the interferences entailed by the scratches through the steps, which will be explained in the following two sections (Section 3.2 and 3.3). Moreover, for the convenience of the following description, we mark the suspected points corresponding to the edges of the rip and scratch (see rip_L, rip_R, scratch_L, and scratch_R in Figure 4b).

### 3.2. Clustering Process

**Figure 5:**
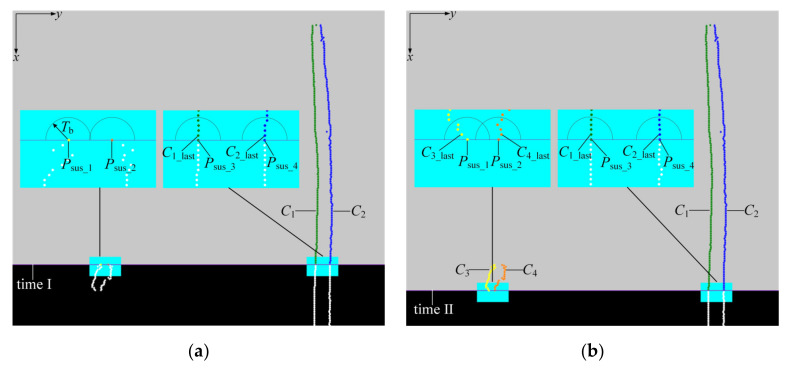

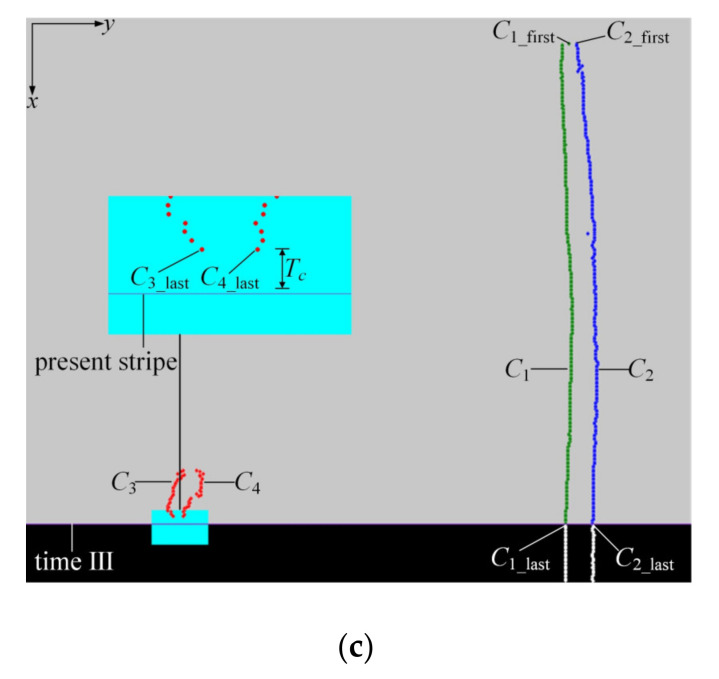
Clustering process and cluster elimination process: (**a**) the result of clustering process at time I. (**b**) The subsequent clustering result of (**a**) after several processing cycles at time II, coloring the points with different colors means the points are clustered in different clusters; (**c**) the result of cluster elimination process at time III, the red points are the invalid data that can be eliminated. In each subfigure, the middle illustrations are the zoom views of the lower little rectangle boxes.

In this step, we separate the suspected points induced by different scratches or rips into different clusters in real time and represent the points in different clusters by coloring them differently in Figure 5.

As shown in Figure 5a, there are four suspected points extracted on the present stripe (denoted by *P*_sus_*i*_, *i* = 1–4) at time I. Moreover, two clusters (denoted by *C_j_*, *j* = 1,2) already exist before time I and the latest point added in cluster *C_j_* is denoted as *C_j_*__last_.

In each processing cycle, three-dimensional Euclidean distance from *P*_sus_*i*_ to every *C_j_*__last_ (denoted by *ρ*(*P*_sus_*i*_,*C_j_*__last_)) is calculated.

(1) If there is a cluster *C_j_* that makes *ρ*(*P*_sus_*i*_,*C_j_*__last_) ≤ *T*_b_, where *T*_b_ is the clustering threshold, then *P*_sus_*i*_ will be added into *C_j_*. Hence, in Figure 5a, *P*_sus_3_ and *P*_sus_4_ are added to clusters *C*_1_ and *C*_2_, respectively. It is worth noting that if there are more than two clusters meeting the condition that *ρ*(*P*_sus_*i*_,*C_j_*__last_) ≤ *T*_b_, then *P*_sus_*i*_ will be added into the one with the smallest Euclidean distance.
(2) If there is no cluster *C_j_* that makes *ρ*(*P*_sus_*i*_,*C_j_*__last_) ≤ *T*_b_, then a new cluster will be created and *P*_sus_*i*_ will be added to it. Thus, we can see that in Figure 5a, new clusters *C*_3_ and *C*_4_ are created and *P*_sus_1_ and *P*_sus_2_ added into them, respectively.

In the next processing cycle, the suspected point *P*_sus_*i*_ that were just added into *C_j_* in present cycle will be treated as new *C_j_*__last_, then this point will be traversed and neighbor searched by the newly suspected points extracted on the next stripe.

Figure 5b is the subsequent clustering result of Figure 5a after several processing cycles. It can be seen that the suspected points on scratch_L, scratch_R, rip_L, and rip_R (see Figure 4b) have been successfully placed into different clusters *C*_3_, *C*_4_, *C*_1_, and *C*_2_ at time II.

The value of the clustering threshold *T*_b_ is determined by the following formula:

(9) $$\left\{\begin{array}{l} T_{b}=\sqrt{S_{x}{}^{2}+S_{y}{}^{2}+S_{z}{}^{2}}\\ S_{x}=v_{\max}/f\\ S_{y}=1.2\text{\textasciitilde{}}1.5\\ S_{z}=1.5\text{\textasciitilde{}}2\; \end{array}\right.$$

where *S*_x_, *S*_y_, and *S*_z_ represent the clustering distances in the *x*, *y*, and *z* directions, respectively. *v*_max_ is the maximum belt speed. *f* is the framerate, namely the number of stripes of input data per second. *S*_x_ denotes the *x*-coordinate difference of the points between two adjacent frames at the maximum belt speed. The values of *S*_y_ and *S*_z_ are determined by analyzing the fluctuation of the points along the rip edges in the *y* and *z* directions, respectively.

### 3.3. Cluster Elimination

In order to ensure the real-time and efficiency of identification, the invalid clusters induced by scratches should be eliminated in time.

It is found that, once the longitudinal rip occurs, the rip will extend infinitely along the *x* direction, and there will be continuous suspected points added into the corresponding clusters. Whereas for the scratches, due to their limited lengths, their edges are scanned in a limited number of processing cycles. Therefore, if no newly suspected points were added to a cluster in recent processing cycles, it indicates that the cluster cannot correspond to a rip, but to a scratch that has been completely scanned, and should be eliminated.

Figure 5c shows that a scratch has been fully scanned at time III and the corresponding suspected points are in *C*_3_ and *C*_4_. The distance from each *C_j_*__last_ to present stripe is calculated by

(10) $$d\left(x_{p},\;C_{j\_\mathrm{last}\text{\_}x}\right)=x_{p}-C_{j\_\mathrm{last}\text{\_}x}$$

where *x*_p_ is the *x*-coordinate value of all points on present stripe and *C _j_*__last_*x*_ is the *x*-coordinate value of point *C_j_*__last_.

Then the cluster *C_j_* and all the points in it will be eliminated, if

(11) $$d\left(x_{p},\;C_{j\_\mathrm{last}\text{\_}x}\right)>T_{c}$$

where *T*_c_ is the distance threshold, which is determined by:

(12) $$T_{c}=s_{c}\cdot v_{\max}/f$$

where *v*_max_ is the maximum belt speed, *f* is the framerate and coefficient *s*_c_ > 1.

After this step, *C*_3_ and *C*_4_ are automatically eliminated because the distances from *C*_3_last_ and *C*_4_last_ to present stripe is more than *T*_c_. By contrary, *C*_1_ and *C*_2_ are retained.

By eliminating the useless clusters, the number of clusters always stays small rather than increasing indefinitely, thus ensuring that the computational time and space costs are relatively low during each processing cycle.

Additionally, the event will be logged when the cluster is eliminated to get the frequency of the scratches. When the frequency of these scratches, which have been scanned, is increased sharply, it indicates that there may be some abnormal situations on the belt. This can be used as a reference for safety inspection, but it does not trigger a longitudinal rip alarm.

### 3.4. Empirical Discrimination

In this step, we realize the real-time identification of the longitudinal rip through an empirical mechanism. As shown in Figure 5c, at time III, the suspected points on rip_L and rip_R have been clustered in *C*_1_ and *C*_2_, respectively. *C_j_*__first_ and *C_j_*__last_ represent the suspected points that firstly and lastly added into the cluster *C_j_*. The longitudinal rip is infinitely extended along the running direction of the belt (the *x* direction) while the size of the scratch is limited in this direction. Thus, longitudinal rip is identified when a cluster grows to a certain size through clustering. We quantify the size of the cluster as

(13) $$g\left(C_{j}\right)=C_{j}{}_{\_\mathrm{last}\_x}-C_{j}{}_{\_\mathrm{first}\_x}$$

where *C_j_*__first_*x*_ and *C_j_*__last_*x*_ denote the *x*-coordinate of point *C_j_*__first_ and *C_j_*__last_, respectively.

Then if *g*(*C_j_*) > *T*_d_, it indicates that the edge corresponding to *C_j_* has been scanned long enough in the *x* direction to be considered as an edge of a longitudinal rip. *T*_d_ is an empirical discrimination threshold, which can be taken as 100 mm, since no scratch is over 100 mm long according to our experimental statistics and survey [3]. On the contrary, once longitudinal rip occurs, the rip length will be far more than 100 mm.

As the belt continues move in the *x* direction, once *g*(*C*_1_) > *T_d_* and *g*(*C*_2_) > *T_d_*, it can be concluded that *C*_1_ and *C*_2_ correspond to the two edges of the longitudinal rip respectively and the longitudinal rip has occurred. Then the system will make intelligent decisions such as alarm and automatic shutdown.

## 4. Phase II: Characterization of the Rip

After the longitudinal rip is identified, we need to make corresponding characterization of the rip to provide intuitive reference information for maintenance staff. Therefore, an effective characterization method used to determine the direction and maximum width of longitudinal rip by PCA (principal component analysis) [27,28] is proposed.

### 4.1. Determination of Rip Direction

**Figure 6:**
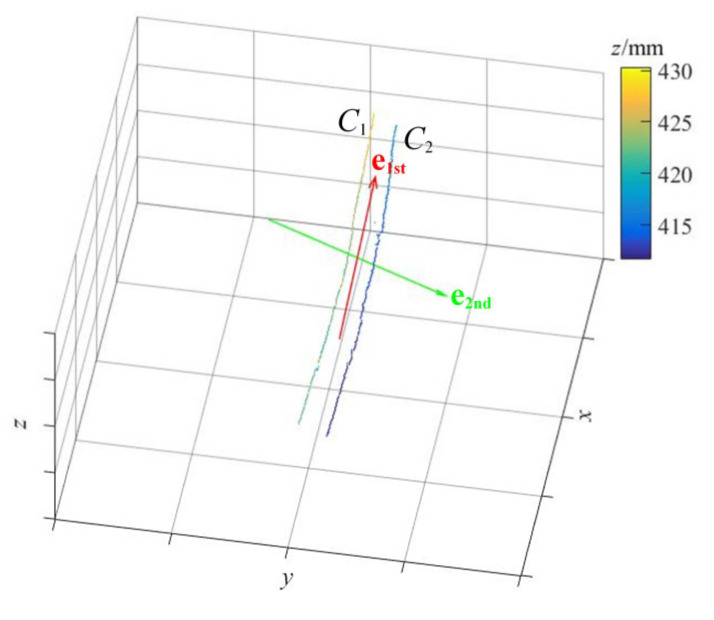
The visualization of the direction of the rip; the color bar corresponds to the *z* coordinate values.

As shown in Figure 6, it is supposed that there are *m* points in cluster *C*_1_ (which correspond to rip_L) and *n* points in cluster *C*_2_ (which correspond to rip_R). All the *m* + *n* rip edge points will be taken out and then form a 3D point cloud matrix **P** of 3 × (*m* + *n*), i.e.,

(14) $$\begin{array}{l} P=[p_{1},\cdots ,p_{m},p_{m+1},\cdots ,p_{m+n}]=[C_{1\_1},\cdots ,C_{1\_m},C_{2\_1},\cdots ,C_{2\_n}]\\ ={\left[\begin{array}{c} C_{1\_1\_x}\\ C_{1\_1\_y}\\ C_{1\_1\_z} \end{array}\cdots \begin{array}{c} C_{1\_m\_x}\\ C_{1\_m\_y}\\ C_{1\_m\_z} \end{array}\begin{array}{c} C_{2\_1\_x}\\ C_{2\_1\_y}\\ C_{2\_1\_z} \end{array}\cdots \begin{array}{c} C_{2\_n\_x}\\ C_{2\_n\_y}\\ C_{2\_n\_z} \end{array}\right]}_{3\times (m+n)} \end{array}$$

Next, the PCA algorithm is used to determine the principal component vectors (**e**_1st_ and **e**_2nd_) of the distribution of **P**:

(1) Firstly, **P** is normalized by the center to get $\tilde{P}$, i.e.,
  (15) $$\left\{\begin{array}{l} \tilde{P}=[{\tilde{p}}_{1},\cdots ,{\tilde{p}}_{m+n}]\\ {\tilde{p}}_{i}=p_{i}-\bar{p},i=1,\cdots ,m+n\\ \bar{p}=\frac{1}{m+n}\sum_{i=1}^{m+n}p_{i} \end{array}\right.$$
(2) Then the covariance matrix **H** is decomposed by singular value decomposition (SVD) [29,30].
  (16) $$H=\tilde{P}{\tilde{P}}^{T}=U\Sigma^{2}U^{T}=[u_{1},u_{2},u_{3}]\left[\begin{array}{ccc} \sigma_{1}^{2} & & \\ & \sigma_{2}^{2} & \\ & & \sigma_{3}^{2} \end{array}\right]\left[\begin{array}{c} u_{1}^{T}\\ u_{2}^{T}\\ u_{3}^{T} \end{array}\right]$$
(3) The principal vectors are the columns of **U**, i.e., **u**_1_, **u**_2_ and **u**_3_. The first principal vector **e**_1st_ is the eigenvector with the largest eigenvalue in **Σ**^2^. Namely, if $\sigma_{i}^{2}$ = max{$\sigma_{1}^{2}$, $\sigma_{2}^{2}$, $\sigma_{3}^{2}$}, then **e**_1st_ = **u***_i_*. Similarly, the second principal vector **e**_2nd_ is the eigenvector with the second largest eigenvalue in **Σ**^2^.

The first principal vector **e**_1st_ calculated by the PCA can represent the length direction of the distribution of the point cloud; therefore, the direction of longitudinal rip can also be represented by **e**_1st_.

### 4.2. Maximum Width of the Rip

**Figure 7:**
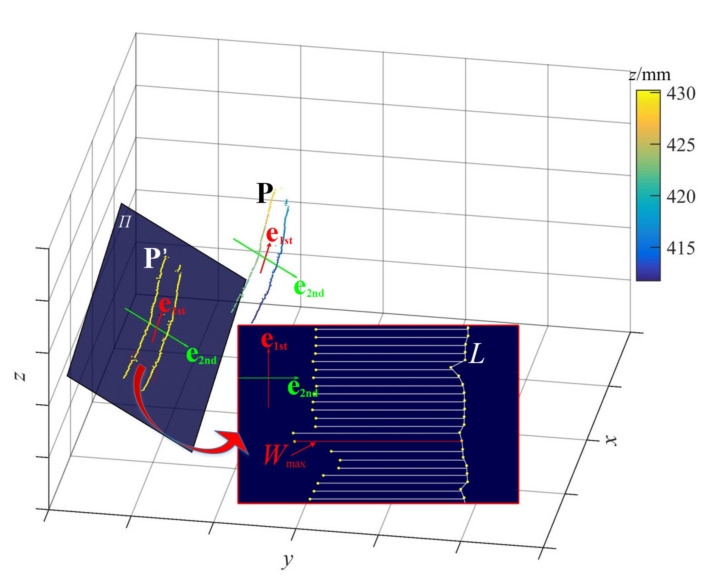
Schematic diagram of rip’s width measurement; the color bar corresponds to the *z* coordinate values; plane *Π* is the projection plane; *W*_max_ is the maximum width of the rip.

The measurement of the maximum width of the longitudinal rip is necessary since it directly reflects the severity of the current tearing situation. However, the measuring method of obtaining the width of the rip by simply calculating the Euclidean distances between the points on the left and right edges of the rip may easily lead to inexact results. As the conveyor belts used in factories and mines (to transport materials) are usually arched, and the rip may occur in any area of the arc. In addition, the direction of the rip is not always parallel to the belt running directions (the *x* direction). Thus, in order to get the more accurate measuring result, we propose a novel characterization model (see Figure 7) to calculate the rip width.

Similar to Section 4.1, PCA is employed to get **e**_1st_ and **e**_2nd_, which, respectively, represent the length direction and width direction of the 3D point cloud distribution (**P**) of the rip.

The 3D points in **P** are projected onto plane *Π,* which is determined by **e**_1st_ and **e**_2nd_. The two-dimensional matrix **P**^’^ of the projection points on *Π* is calculated by:

(17) $$\begin{array}{l} P^{'}=[p_{1}^{'},\cdots ,p_{m}^{'},p_{m+1}^{'},\cdots ,p_{m+n}^{'}]=\left[\begin{array}{c} e_{1\mathrm{st}}^{T}\\ e_{2\mathrm{nd}}^{T} \end{array}\right]P\\ =\left[\begin{array}{c} e_{1\mathrm{st}}^{T}\\ e_{2\mathrm{nd}}^{T} \end{array}\right]{\left[\begin{array}{c} C_{1\_1\_x}\\ C_{1\_1\_y}\\ C_{1\_1\_z} \end{array}\cdots \begin{array}{c} C_{1\_m\_x}\\ C_{1\_m\_y}\\ C_{1\_m\_z} \end{array}\begin{array}{c} C_{2\_1\_x}\\ C_{2\_1\_y}\\ C_{2\_1\_z} \end{array}\cdots \begin{array}{c} C_{2\_n\_x}\\ C_{2\_n\_y}\\ C_{2\_n\_z} \end{array}\right]}_{3\times (m+n)} \end{array}$$

where **$p_{1}^{\prime }$**, …, $p_{m}^{\prime }$ are 2D points transformed from the left edge points (rip_L) of the rip and $p_{m+1}^{\prime }$, …, $p_{m+n}^{\prime }$ are from the right edge (rip_R).

Then the adjacent points among **$p_{m+1}^{\prime }$** – **$p_{m+n}^{\prime }$** are connected to form a polyline *L*. Points **$p_{1}^{\prime }$** − **$p_{m}^{\prime }$** are taken as the starting points and vector **e**_2nd_ is taken as the direction to make half-lines. These half-lines will intersect the polyline *L*, and the distance between each intersection point and the starting point is calculated. Then the maximum width *W*_max_ of the rip can be represented by the maximum distance.

## 5. Experiment Validation

### 5.1. Experiment System Building

**Figure 8:**
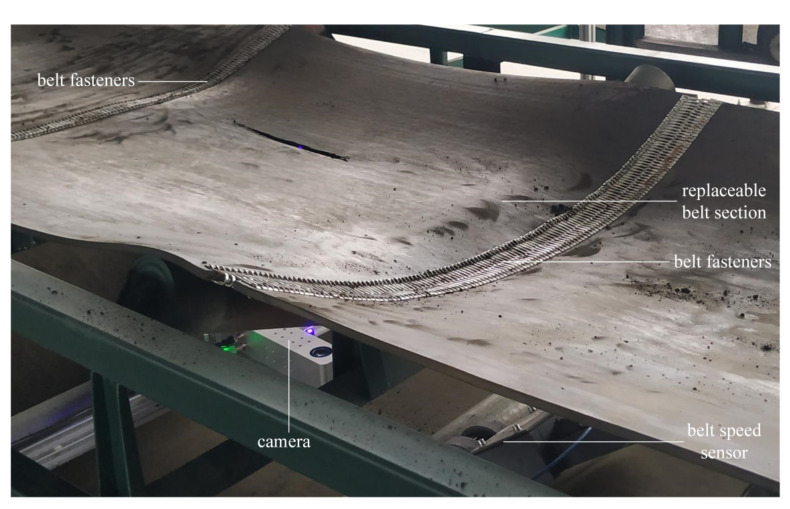
The simulation experiment platform in laboratory; only the replaceable belt section needs to be replaced rather than the whole belt in each experiment.

As shown in Figure 8, we built a simulation experimental platform in the laboratory. The trough conveyor widely used in factories and mines [31] is selected as the experimental conveyor, which adds the practical value of this study and also makes the result more representative. The trough angle is 20° and the belt width is 1 m. Moreover, we make 1 × 1 m belt sections into a replaceable form by installing belt fasteners at both ends of them so that different experiment data can be collected by replacing the replaceable belt sections rather than the whole belt.

The camera used in the experiment is a binocular line laser stereo vision camera (VZ-XJGY-1300G) produced by Vizum corporation (Beijing, China). This camera can uniformly collect 2000 3D point coordinates on each laser stripe and acquire the data from 1000 stripes per second. An IPC with Core i7 6700 CPU, NVIDA GTX 1050Ti Graphics card, 8 GB memory, and a Windows 10 operating system were used in this experiment to process data. The IPC communicates with the camera through a 1000 M network cable. We programed the experiment procedure using Visual C++ to verify the proposed algorithm and the parameters were set as follows: the extraction threshold in *z T*_az_ = 12, the extraction threshold in *y T*_ay_ = 3 mm, the clustering threshold *T*_b_ = 3.67 mm, the distance threshold *T*_c_ = 3 mm, the discrimination threshold *T*_d_ = 100 mm.

Twenty belt sections were selected as the experimental samples, and 10 of them were taken from the new belts and the other 10 were taken from the old belts. On these 20 belt sections, the rips were artificially created. These belt sections were replaced on the conveyor one-by-one to test the identification and characterization method in this work.

### 5.2. Experimental Results

By using the identification algorithm proposed in Section 3, we performed 50 tests on each belt section at different speeds (10 tests at 0.5, 1.0, 1.5, 2.0, and 2.5 m/s, respectively) and obtained good identification results. The correct rate was 99.2% and the identification times (from the time when the 100 mm rip length in *x* direction was scanned to the time when the identification result was obtained) were less than 0.04 ms. Furthermore, the calculation results of the rip’s maximum width *W*_max_ were obtained by the characterization method proposed in Section 4 and the relative errors are within ±5%.

**Figure 9:**
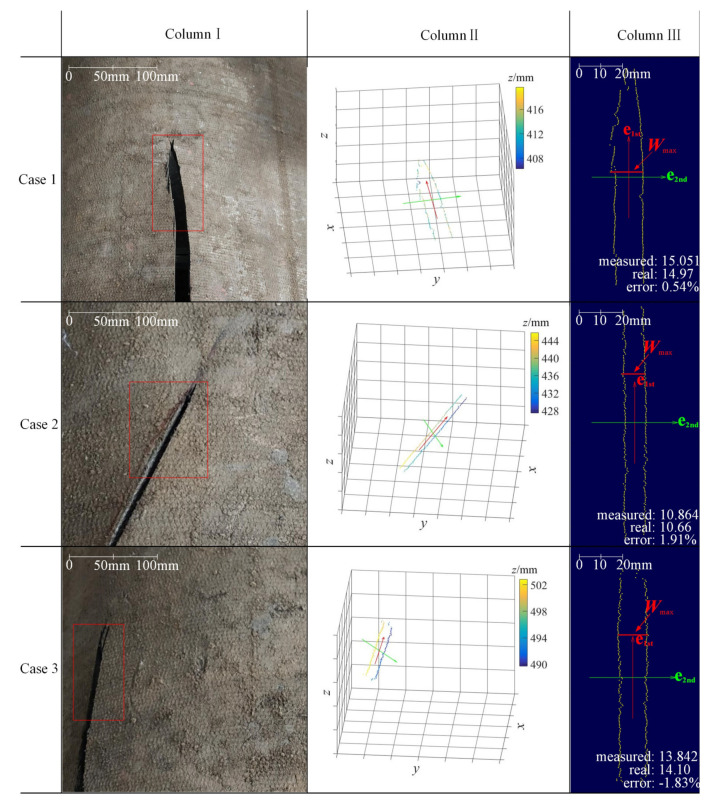
The experimental results; column I: physical pictures; column II: the 3D visualization of the rip edge points, the color bar corresponds to the *z* coordinate values; column III: the calculation result visualization of max width of each rip.

To further demonstrate the effectiveness of the method, Figure 9 shows the experimental results of three representative cases. Case 1 is a general case where the rip is located in the center of the belt and its direction is almost parallel to the running direction (the *x* direction). Case 2 and Case 3 are two extreme cases; one is that the rip direction and the running direction are at a relatively large angle; the other is that the rip is close to the side of the belt. In each case, when a certain length (100 mm in this work) of the rip was scanned (see the red rectangle boxes in Column I), the identification result would be obtained within 0.04 ms. In Column II, we can see that these rips are accurately identified and the longitudinal rip edge points are accurately extracted. Using the characterization method, the max width of each rip is obtained in the 2D coordinate system determined by the principal vectors **e**_1st_ and **e**_2nd_ (see Column III). This indicates that the identification and characterization method proposed in this work is suitable for different situations.

**Figure 10:**
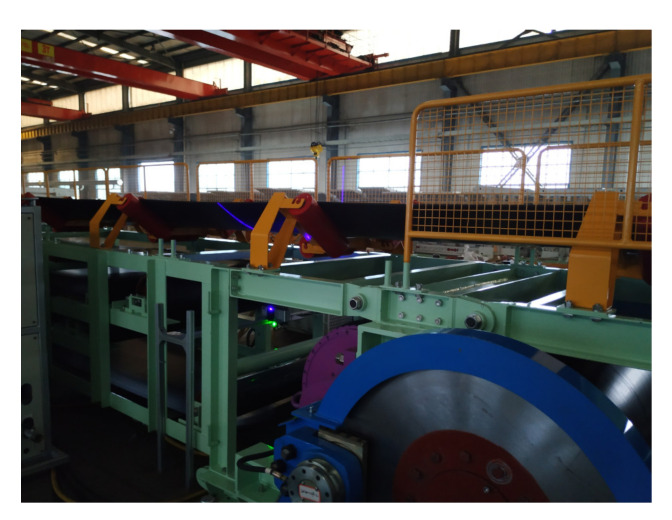
The experiment in industrial scene.

To further test the proposed method, we set up the identification system on a belt conveyor, located at Shandong Energy Reshipment Group Co., Ltd in China (see Figure 10).

**Figure 11:**
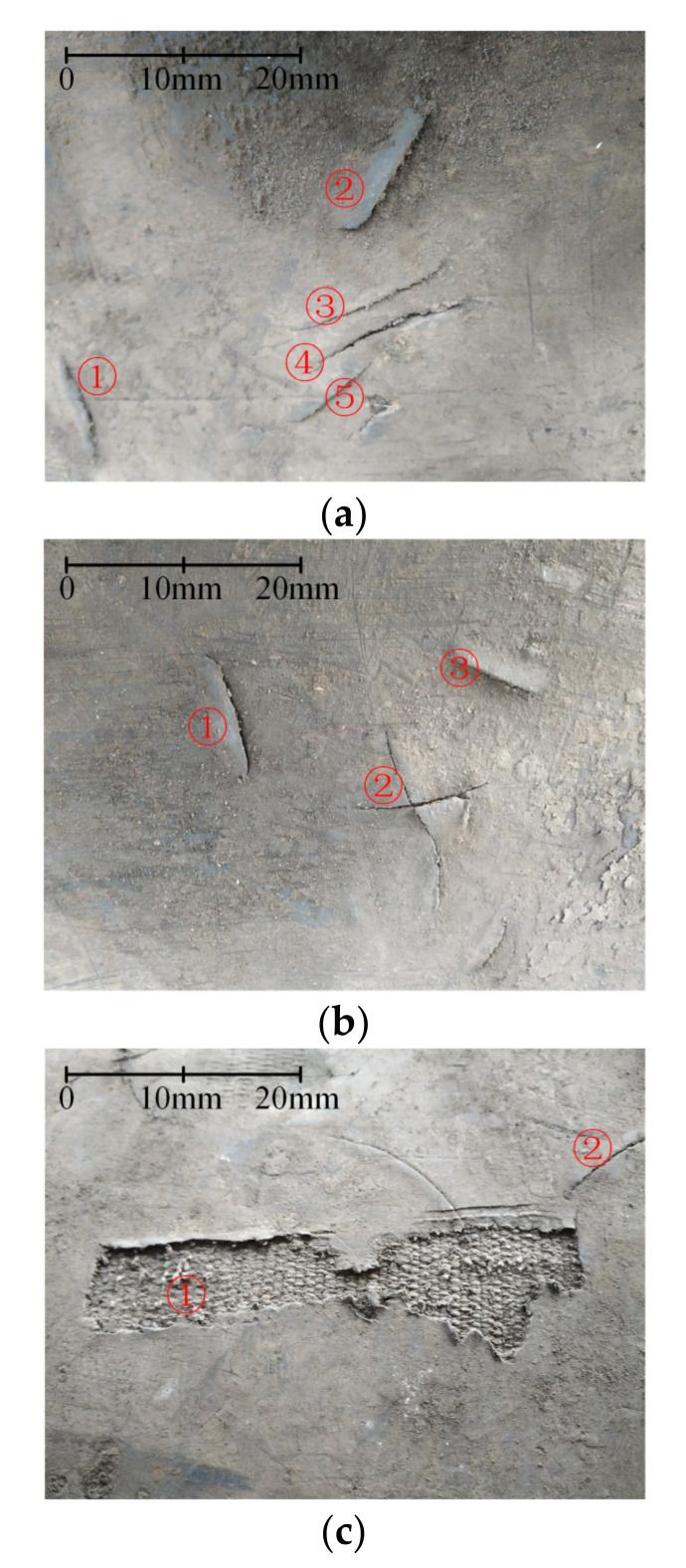
The scratches on the lower surface of the belt. There are five scratches in (**a**), three in (**b**), and two in (**c**).

The site environment was dim and dusty. The conveyor belt width was 1.4 m and the conveyor would vibrate when running. During the 48 hours of the system’s operation in the industrial scene, no longitudinal rip occurred. Nevertheless, we found many different scratches on the lower surface of the belt. As shown in Figure 11, there were five scratches in Figure 11a, three in Figure 11b, and two in Figure 11c.

Using the traditional methods based on image processing, these scratches can easily be confused with longitudinal rips. However, using the method in this work based on 3D point cloud processing, the effects of lighting conditions and stains on the belt surface will be eliminated. In addition, since the scanned length of all the scratches would not exceed 100 mm, the scratches in the experiment would not trigger the condition mentioned in Section 3.4: the length of a defect scanned along the *x* direction being longer than the distance threshold *T*_d_ (100 mm in this work). Therefore, the identifying system herein makes it possible to effectively differentiate rips from scratches, which avoids the false alarm and unplanned downtime. Meanwhile, the information of the scratches was recorded, which can be further used as a reference for safety inspection.

## 6. Conclusions

To summarize, to the best of our knowledge, this is the first time a belt longitudinal rip detection and characterization method based on 3D point cloud processing was proposed; it could work in a harsh environment. Using a binocular line laser stereo vision camera, the 3D point cloud data on the lower surface of the belt was collected in a line-scanning mode. The proposed identification algorithm was used in each processing cycle to process the 3D point cloud data and identify the longitudinal rip in real time. The experimental results show that the proposed method is effective at identifying longitudinal rips whose widths are more than 3 mm. The issue of a false alarm caused by the scratches was solved perfectly by using this method and the identification correct rate was 99.2% in all experiments we performed. Meanwhile, compared with the time required by image processing methods for longitudinal rip detection (about 18–50 ms) [19,32,33], the identification time of this method was greatly shorter (0.01–0.04 ms). Furthermore, the PCA algorithm was employed to realize the effective characterization of the identified rip, and the relative error of the calculation result of the rip’s maximum width was within ±5%. Compared with the characterization method based on 2D image processing [18], the proposed method realized 3D characterization for the longitudinal rips; hence, it is more applicable and has higher precision. This method is suitable for trough belt conveyors with belt widths of less than 1.4 m, and can be used in mines, ports, power plants, and other occasions. In order to make the proposed method in this work have higher application value and reliability, we will perform more long-term tests in a variety of industrial scenes and multiform conveyors (e.g., pipe conveyors) to further verify the method. In addition, it should be noted that the method in this work could not only could be used for the identification and characterization of the belt longitudinal rip, but it also has broad application prospects in solving other defect detection problems, such as defect detection for mechanical parts, buildings, roads, tracks, etc.

## Data Availability

The simulation and experimental data used to support the findings.

## Appendix A

**Table A1:** List of symbols in this paper.

Symbol	Notes
*b*/mm	The thickness of the belt
*C_j_*	The *j*-th cluster
*C_j_*__first_	The firstly suspected point added in cluster *Cj*
*C_j_*__last_	The latest suspected point added in cluster *Cj*
*C _j_*__last_*x*_/mm	The *x*-coordinate value of point *C_j_*__last_
**e**_1st_	The first principal vector of P
**e**_2nd_	The second principal vector of P
*f*	Framerate, the number of stripes of input data per second
**P**	The 3D point cloud matrix of the rip edge points
*P*_sus_*i*_	The *i*-th suspected point extracted on the present stripe
*s*_a_	The empirical coefficient to determine *T*_az_
*s*_c_	The coefficient to determine *T*_c_
*S*_x_/mm, *S*_y_/mm, *S*_z_/mm	The coefficient to determine *T*_b_
*T*_ay_/mm	The extraction threshold of space in *y* direction
*T*_az_	The extraction threshold of change rate in *z* direction
*T*_b_/mm	The clustering threshold
*T*_c_/mm	The distance threshold
*T*_d_/mm	The empirical discrimination threshold
*t_i_*/s	The timestamp to get points on the *i*-th laser stripe
*v*/(mm/s)	The real-time belt speed
*v*_max_/(mm/s)	The maximum belt speed
*W*_max_/mm	The maximum width of the rip
*x_j_*/mm	The coordinate value in the belt running direction of the *j*-th point on the laser stripe from left to right
*x*_p_/mm	The *x*-coordinate value of all points on present stripe
*y_j_*/mm	The coordinate value in the width direction of the *j*-th point on the laser stripe from left to right
*y*_max_/mm	The largest *y* coordinate value among all points on each laser stripe
*y_min_*/mm	The smallest *y* coordinate value among all points on each laser stripe
*z_j_*/mm	The coordinate value in the height direction of the *j*-th point on the laser stripe from left to right
